# C-reactive protein lowers the serum level of IL-17, but not TNF-α, and decreases the incidence of collagen-induced arthritis in mice

**DOI:** 10.3389/fimmu.2024.1385085

**Published:** 2024-04-08

**Authors:** Sanjay K. Singh, Amanda Prislovsky, Donald N. Ngwa, Undral Munkhsaikhan, Ammaar H. Abidi, David D. Brand, Alok Agrawal

**Affiliations:** ^1^Department of Biomedical Sciences, James H. Quillen College of Medicine, East Tennessee State University, Johnson City, TN, United States; ^2^The Lt. Col. Luke Weathers, Jr. VA Medical Center, Memphis, TN, United States; ^3^College of Dental Medicine, Lincoln Memorial University, Knoxville, TN, United States

**Keywords:** C-reactive protein, collagen-induced arthritis, IL-17, immune complex, TNF-α

## Abstract

The biosynthesis of C-reactive protein (CRP) in the liver is increased in inflammatory diseases including rheumatoid arthritis. Previously published data suggest a protective function of CRP in arthritis; however, the mechanism of action of CRP remains undefined. The aim of this study was to evaluate the effects of human CRP on the development of collagen-induced arthritis (CIA) in mice which is an animal model of autoimmune inflammatory arthritis. Two CRP species were employed: wild-type CRP which binds to aggregated IgG at acidic pH and a CRP mutant which binds to aggregated IgG at physiological pH. Ten CRP injections were given on alternate days during the development of CIA. Both wild-type and mutant CRP reduced the incidence of CIA, that is, reduced the number of mice developing CIA; however, CRP did not affect the severity of the disease in arthritic mice. The serum levels of IL-17, IL-6, TNF-α, IL-10, IL-2 and IL-1β were measured: both wild-type and mutant CRP decreased the level of IL-17 and IL-6 but not of TNF-α, IL-10, IL-2 and IL-1β. These data suggest that CRP recognizes and binds to immune complexes, although it was not clear whether CRP functioned in its native pentameric or in its structurally altered pentameric form in the CIA model. Consequently, ligand-complexed CRP, through an as-yet undefined mechanism, directly or indirectly, inhibits the production of IL-17 and eventually protects against the initiation of the development of arthritis. The data also suggest that IL-17, not TNF-α, is critical for the development of autoimmune inflammatory arthritis.

## Introduction

C-reactive protein (CRP) circulates in the blood and is deposited at sites of inflammation (1). CRP is composed of five identical subunits arranged in a cyclic pentameric symmetry and whose production and secretion by hepatocytes are increased in inflammatory states (2–4). CRP is a dual pattern recognition molecule; however, which pattern is recognized by CRP depends upon its pentameric structural conformation (5). In the plasma and elsewhere where CRP is present in its native conformation, CRP binds to phosphocholine (PCh)-containing substances and subsequently activates the classical pathway of complement (6–9). It has been suggested that the native pentameric conformation of CRP is subtly altered at sites of inflammation where an inflammatory milieu of acidic pH and redox condition is present (10–13). Such non-native pentameric CRP recognizes an additional pattern and that is amyloid-like structures exposed on immobilized proteins (5). Other mechanisms for the alteration of the native conformation of CRP have also been reported (14, 15). It has also been shown that the combination of the two recognition functions of CRP in two different pentameric conformations contributes to the protection against diseases caused by the deposition of otherwise fluid-phase proteins, such as pneumococcal infection (16, 17). Additionally, it has been hypothesized that the pathogen-defense functions of CRP are preserved even when the PCh-binding site of CRP is blocked by a PCh-mimicking compound (18). Non-native pentameric CRP may eventually lead to the generation of CRP monomers (mCRP) which shares the immobilized protein ligand-binding properties and antigenic epitopes of non-native pentameric CRP (1, 10, 19–22). The PCh-binding activity, however, is either retained, decreased or abolished in mCRP depending upon the method of generation of mCRP (23–25).

The changes in the structure of native pentameric CRP caused by acidic pH are reversible at physiological pH (10). Therefore, acidic pH-modified CRP cannot be administered into animal models of human diseases to explore the functions of non-native pentameric CRP *in vivo*. Accordingly, CRP mutants created by site-directed mutagenesis and which mimic the ligand-binding properties of acidic pH-modified native CRP have been employed in *in vivo* experiments (16, 17). One such CRP mutant is Y40F/E42Q CRP in which Tyr40 and Glu42 have been substituted with Phe and Gln, respectively (16, 26). Like acidic pH-treated native CRP, the Y40F/E42Q CRP mutant recognizes both PCh and amyloid-like structures exposed on immobilized proteins (5). The only difference in the functions of native CRP and mutant CRP is that, at physiological pH, native CRP recognizes PCh while mutant CRP recognizes both PCh and amyloid-like structures (5, 16, 26, and data not shown).

Rheumatoid arthritis (RA) is a joint disease characterized by the presence of immune complexes (ICs) in the joints (27, 28). The synovium of the RA joints has an inflammatory milieu characterized by acidic pH and redox conditions (29–32). CRP is also deposited in the synovium (33), raising the possibility that CRP is present in the synovium in both, native and non-native, pentameric conformations. It has also been shown that the serum levels of IL-17, IL-6, TNF-α, IL-10, IL-2 and IL-1β are altered in RA patients (28, 34, 35). CRP is used as a nonspecific biomarker of inflammation during the development of RA (3, 36). Previously published reports suggest that pentameric CRP protects against the development of inflammatory arthritis in mice; however, the mechanism of action of CRP *in vivo* including the effects of CRP on the production of cytokines *in vivo* are not fully elucidated yet (37–39).

In this study, we administered wild-type (WT) CRP and the CRP mutant Y40F/E42Q in mice with collagen-induced arthritis (CIA) and monitored the development of the disease and measured the serum levels of IL-17, IL-6, TNF-α, IL-10, IL-2 and IL-1β. The CIA mouse model is a model of autoimmune inflammatory arthritis that shares many clinical features with human RA (40). We tested the hypothesis that CRP changes its structure in the synovium, binds to immobilized ICs, and subsequently protects against the development of arthritis. Accordingly, we also hypothesized that the CRP mutant might be more protective than WT CRP against the development of CIA.

## Materials and methods

### Preparation of aggregated IgG

Aggregated IgG (agg-IgG) was prepared according to a published method (41). In brief, human IgG (Sigma, I4506) at 10 mg/ml in normal saline was heated at 63°C for 20 min in a shaking water bath. After centrifugation at 10,000 rpm for 5 min, the precipitate was discarded. The concentration of IgG in the supernatant containing agg-IgG was determined by measuring the absorbance at 280 nm and adjusted to a final concentration of 1 mg/ml of agg-IgG. This preparation of agg-IgG was used as a model of ICs (42) to evaluate the binding of CRP to immobilized ICs.

### Detection of amyloid-like structures on immobilized IgG and agg-IgG

The presence of amyloid-like structures on immobilized IgG was detected as described previously (5). Microtiter wells (Corning, 9018) were coated with 10 μg/ml of monomeric IgG (mono-IgG) or agg-IgG in TBS, pH 7.2, and incubated at 4°C overnight. The unreacted sites in the wells were blocked with TBS containing 0.5% gelatin at room temperature for 45 min. Both, polyclonal antibodies (Novus, NBP2-25093) and monoclonal antibodies (Novus, NBP2-13075) to amyloid-β peptide 1-42 (Aβ) were used to detect the amyloid-like structures formed on the mono-IgG and agg-IgG following their immobilization. Normal rabbit IgG and normal mouse IgG were used as controls for the antibodies. The antibodies (10 μg/ml) diluted in TBS containing 0.1% gelatin and 0.02% Tween 20 were added to the wells and incubated at 37°C for 1 h. After washing the wells, bound polyclonal anti-Aβ antibodies were detected by using HRP-conjugated donkey anti-rabbit IgG (GE Healthcare) and bound monoclonal anti-Aβ antibodies were detected by using HRP-conjugated goat anti-mouse IgG (Thermo Fisher Scientific). Color was developed with ABTS reagent and the OD was read at 405 nm in a microtiter plate reader (Molecular Devices). Immobilized Aβ peptide 1-42 (Bachem) was used as a control for immobilized IgG and for anti-Aβ antibodies.

### Preparation of CRP

Native WT CRP was purified from discarded human pleural fluid as described previously (43). Recombinant mutant CRP Y40F/E42Q was expressed in CHO cells using the ExpiCHO Expression System (Thermo Fisher Scientific) and purified from the culture supernatant exactly as described for WT CRP (16). The construction of the Y40F/E42Q mutant CRP cDNA has been reported previously (26). In brief, WT and mutant CRP were purified by Ca^2+^-dependent affinity chromatography on a PCh-conjugated Sepharose column, followed by ion-exchange chromatography on a MonoQ column and gel filtration on a Superose12 column. Purified CRP was dialyzed against TBS, pH 7.2, containing 2 mM CaCl_2_, and was subsequently treated with Detoxi-Gel Endotoxin Removing Gel (Thermo Fisher Scientific) according to manufacturer’s instructions. The concentration of endotoxin in CRP preparations was determined by using the Limulus Amebocyte Lysate kit QCL-1000 (Lonza). The concentration of endotoxin in WT and mutant CRP preparations were 0.52 ± 0.06 EU/50 μg and 0.60 ± 0.07 EU/50 μg, respectively. Purified CRP in TBS, pH 7.2, containing 2 mm CaCl_2_, was stored at 4°C, and was used within a week.

### CRP-IgG binding assay

The CRP-IgG binding assays were performed as described previously (12). In brief, microtiter wells (Corning, 9018) were coated with 10 μg/ml of mono-IgG or agg-IgG diluted in TBS, pH 7.2, and incubated at 4°C overnight. The unreacted sites in the wells were blocked with TBS containing 0.5% gelatin. Mutant CRP was diluted in TBS, pH 7.2, containing 0.1% gelatin, 0.02% Tween 20, and 2 mm CaCl_2_ (TBS-Ca) and added to the wells. WT CRP was diluted in TBS-Ca at pH 7.2 and also at pH 5.0 and added to the wells. The plate was then incubated at 37°C for 2 h. After washing the wells, HRP-conjugated goat anti-human CRP antibody (Alpha Diagnostic International, Cat # CRP-11-HRP) was used (5 μg/ml, 37°C, 1 h) to detect bound WT and mutant CRP. Color was developed with ABTS reagent and the OD was read at 405 nm in a microtiter plate reader.

### CIA in mice

CIA was induced in DBA/1 mice as previously described (40). Eight-week-old female DBA/1J mice (Jackson Laboratories, stock# 000670) were used in experiments according to protocols approved by and conducted in accordance with the guidelines administered by the Institutional Animal Care and Use Committee of the Memphis VA Medical Center. Following acclimatization of mice for one week, arthritis was induced by immunization of mice with bovine type II collagen extracted and purified in the investigator (DDB’s) laboratory and emulsified 1:1 in complete Freund’s adjuvant, which was also prepared freshly in DDB’s laboratory.

Two different regimens were employed to evaluate the effects of passively administered WT and mutant CRP on the development of CIA in mice. In regimen 1, the administration of CRP began (day 26) about three weeks after immunization (day 7) and two weeks prior to the onset of the disease (day 40), and the injection of CRP continued till day 44. In regimen 2, the administration of CRP began (day 35) four weeks after immunization (day 7) and on the day of the onset of the disease (day 35), and the injection of CRP continued till day 53. In both regimens, a total of ten CRP injections (50 μg/injection) were given intravenously on alternate days. The control group of mice was administered with TBS, the vehicle for CRP.

The development of arthritis in the fore and hind paws was monitored by visual inspection as described previously (40). Each paw could receive an arthritis score from 0 to 4, and the maximum score could reach 16 per mouse for four paws. Clinical scores were assessed every third day in a blinded manner for each paw, as follows: score 0, normal; score 1, one paw joint affected or minimal diffuse erythema and swelling; score 2, two paw joints affected or mild diffuse erythema and swelling; score 3, three paw joints affected or moderate diffuse erythema and swelling; score 4, all four-digit joints affected or severe diffuse erythema and severe swelling of the entire paw, unable to flex digits.

Four parameters were evaluated: 1. Incidence of arthritis which reflects the percentage of mice that were positive for arthritis. 2. Arthritic limbs/arthritic mouse which reflects the number of arthritic limbs in each arthritic mouse. 3. Clinical score of the severity of arthritis (1-4)/arthritic limb: Scores from all limbs in each arthritic mouse/number of arthritic limbs. 4. Clinical score of the severity of arthritis (1-16) (4 limbs and each 1-4) of all 4 limbs combined/arthritic mouse: Scores from all limbs in all arthritic mice/Number of arthritic mice. The statistical analyses of the data were performed by employing linear regression analysis of the slopes and nonparametric Mann-Whitney test using the GraphPad Prism 9 software and as described in detail in the figure legends.

### Measurement of cytokines

To measure the serum levels of cytokines (IL-17, IL-6, TNF-α, IL-10, IL-2, and IL-1β), sera were collected from mice used in the experiment involving regimen 1. Mice used in the experiment involving regimen 2 were not analyzed for cytokines. On day 26, mice were divided into three groups, and the administration of either WT CRP, mutant CRP, or vehicle began. The injections continued till day 44; the disease onset was on day 40. Cytokines were measured in the sera collected on days 1, 19 and 47. The serum samples were thawed and brought to room temperature, then centrifuged before use. The cytokine levels were measured using Mesoscale Discovery (MSD) Mouse U-PLEX Custom Biomarker Multiplex Assays from Mesoscale Diagnostics, (Maryland, USA). U-Plex assays use biotinylated capture antibodies that are specific for each analyte and MSD employs an electrochemiluminescence technique that allows for the multiplexing. Samples (10 µl) were transferred onto a pre-coated MSD with 10 µl diluent (2-fold dilution) per manufacturer’s guidelines for cytokines IL-17, IL-6, TNF-α, IL-10, IL-2, and IL-1β. The samples were then incubated on a shaker for 2 h at room temperature, followed by washing and the addition of SULFO-TAG detection antibodies. The detection antibodies were incubated for another 2 h, and washed and removed from the plate. 150 µl of 2x MSD read buffer was added and the U-Plex plate and the plate were immediately loaded onto the MSD instrument. Mesoscale SQ120 and SECTOR Imager SI 2400A were used to read the plates according to the manufacturer’s instructions. The intensity of light emitted was then quantified, which was proportional to the sample analyte present. A five-parameter logistic regression method was used to calculate sample concentration and standard curves.

The scatter plots of the data and the median values of the concentrations of cytokines in each group were generated using the GraphPad Prism 9. To determine *p*-values for the differences in the level of each cytokine among various groups at each time point, scatter plots were compared using the software’s Mann-Whitney test which included all the dots in the scatter plots and not just the median values for each time point.

## Results

### Immobilized IgG expresses amyloid-like structures

**Figure 1:**
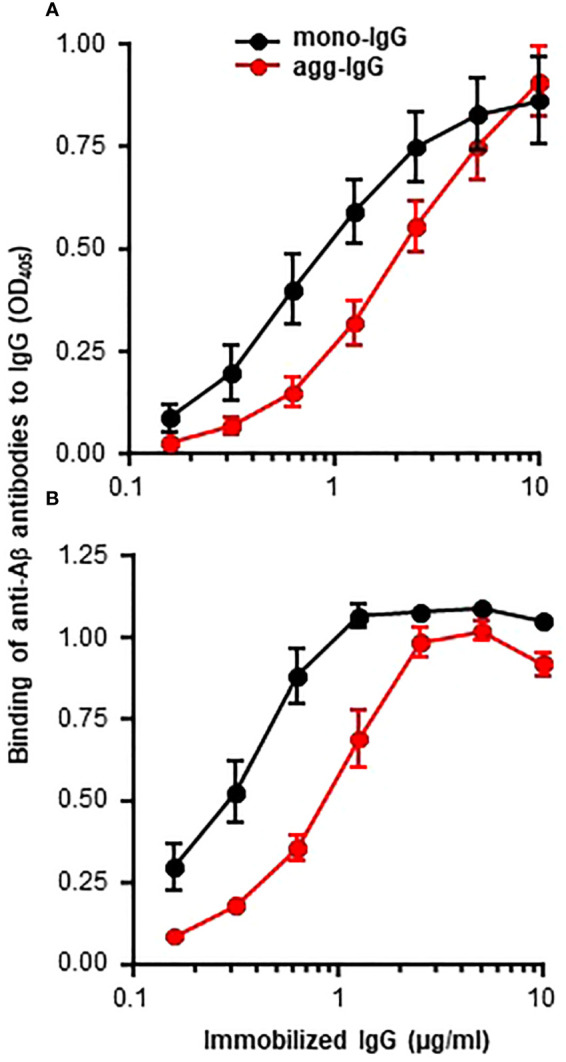
Binding of anti-Aβ antibodies to immobilized IgG. Microtiter wells were coated with mono-IgG and agg-IgG. **(A)** Monoclonal anti-Aβ antibodies were added to the wells to detect the amyloid-like structures formed on immobilized IgG. Bound antibodies were detected by using HRP-conjugated goat anti-mouse IgG. **(B)** Polyclonal anti-Aβ antibodies were added to the wells to detect the amyloid-like structures formed on immobilized IgG. Bound antibodies were detected by using HRP-conjugated donkey anti-rabbit IgG. Data shown are mean ± SEM of three experiments.

It was reported recently that some proteins, when immobilized on microtiter plates, express amyloid-like structures that can be detected by using anti-Aβ antibodies (5). In this study, we investigated whether immobilized IgG also expresses amyloid-like structures by employing normal human mono-IgG and agg-IgG coated on microtiter wells. Both mono-IgG and agg-IgG were immobilized; although, immobilization itself can cause aggregation of mono-IgG on the wells. Two different anti-Aβ antibodies were used to identify the expression of amyloid-like structures on immobilized IgG. We have previously reported the authentication of these antibodies using purified Aβ peptides (5). As shown in **Figure 1**, both immobilized mono-IgG and agg-IgG reacted with both monoclonal (**Figure 1A**) and polyclonal anti-Aβ antibodies (**Figure 1B**). These data suggested that immobilized IgG expressed Aβ epitopes, suggesting that immobilized ICs might also express Aβ epitopes.

### CRP mutant Y40F/E42Q binds to immobilized IgG at physiological pH

**Figure 2:**
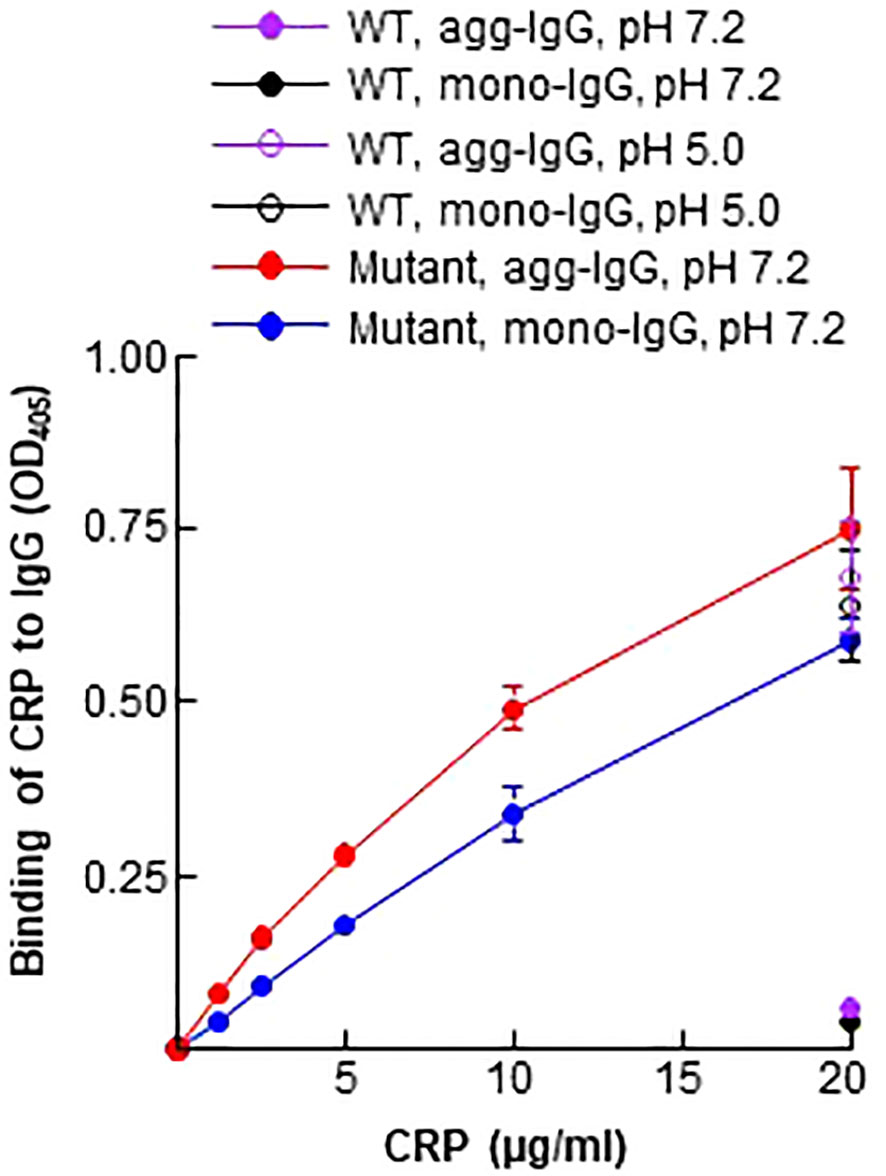
Binding of CRP to immobilized IgG. Microtiter wells were coated with mono-IgG and agg-IgG. WT CRP (a single concentration of 20 μg/ml), diluted in TBS-Ca, pH 7.2 and pH 5.0, was added to the wells. Mutant CRP (two-fold serial dilutions of 20 μg/ml) was diluted in TBS-Ca, pH 7.2, and was added to the wells. Bound CRP was detected by using HRP-conjugated goat anti-human CRP antibodies. Data shown are mean ± SEM of three experiments. Due to low SEM, the error bars are not visible for all the data points in the graph.

Since Aβ is a ligand of the CRP mutant Y40F/E42Q used in this study (5), and since immobilized IgG expresses Aβ epitopes (**Figure 1**), we determined whether the CRP mutant can bind to immobilized IgG at physiological pH. WT CRP at pH 5.0 and pH 7.2 were included as positive and negative controls for the binding of mutant CRP to immobilized IgG. As shown in **Figure 2**, and as has been reported previously (5), WT CRP did not bind to either mono-IgG or to agg-IgG at physiological pH, but, at acidic pH, WT CRP bound to immobilized IgG. The CRP mutant, however, bound to both mono-IgG and agg-IgG at physiological pH in a CRP concentration-dependent manner.

### CRP decreases the incidence of arthritis

**Figure 3:**
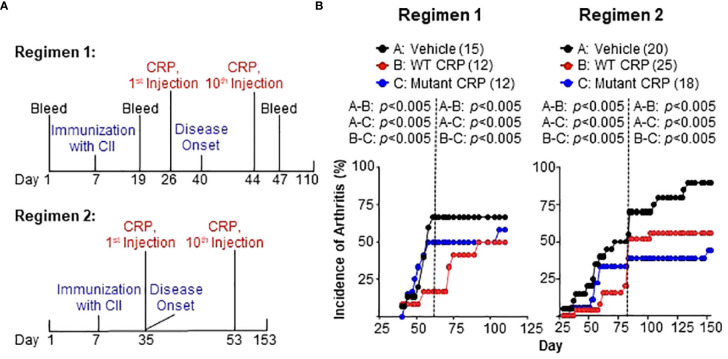
**(A)** Regimens for administering CRP. In regimen 1, CRP administration began two weeks prior to the onset of the disease, while in regimen 2, the CRP administration began on the same day as the onset of the disease. A total of ten CRP injections were given in both regimens. In regimen 1, the disease was monitored for 110 days while in regimen 2 the disease was monitored for 153 days. **(B)** Incidence of arthritis in mice from regimen 1 (left panel) and regimen 2 (right panel). The number of mice in each group is shown in the parentheses. For statistical analyses of the data, the curves in both panels were divided into two parts as shown by the dotted vertical lines. For regimen 1, the curves were divided into days 40-61 and 62-110. For regimen 2, the curves were divided into days 28-83 and 84-153. For the time period of 40-61 days in regimen 1 and 28-83 days in regimen 2, *p* values were determined by employing linear regression analysis of the slopes. For the time period of 62-110 days in regimen 1 and 84-153 days in regimen 2, *p* values were determined by employing Mann-Whitney test. The *p* values for the differences between groups A and B, groups A and C, and groups B and C are shown (all *p* < 0.005).

Two different regimens were employed to evaluate the effects of CRP on the development of CIA (**Figure 3A**). In regimen 1, the administration of CRP (on day 26) began three weeks after immunization with CII (on day 7) and two weeks prior to the onset of the disease (on day 40), and the administration of CRP was continued until day 44. In regimen 2, the administration of CRP (on day 35) began four weeks after immunization (on day 7) and on the day of the onset of the disease (day 35), and the administration of CRP was continued until day 53. CRP was administered on alternate days between days 28-46 in regimen 1 and between days 35-53 in regimen 2.

The effects of CRP on the incidence of arthritis for both regimens are shown in **Figure 3B**. In regimen 1, the data were analyzed separately for days 40-61 and days 62-110 (**Figure 3B**, left), while in regimen 2 (**Figure 3B**, right), the data were analyzed separately for days 28-83 and days 84-153. In both regimens, and in both segments of the data, there were statistically significant differences between vehicle and WT CRP, between vehicle and mutant CRP, and between WT and mutant CRP.

In regimen 1, WT CRP was more protective than mutant CRP in reducing the incidence of CIA. The most dramatic difference in the incidence was around day 60 when the incidence was 72% in the vehicle group compared to 50% and 20% in mutant CRP-treated and WT CRP-treated groups, respectively. In regimen 2, during days 28-83, WT CRP was more protective than mutant CRP as in regimen 1; however, during days 84-153, mutant CRP was more protective than WT CRP. The most dramatic difference in the incidence was around day 80 when the incidence was 75% in the vehicle group compared to 60% and 40% in mutant CRP-treated and WT CRP-treated groups, respectively.

Thus, both regimens gave similar results for incidence; it did not matter whether there was only one injection of CRP or whether there were six injections of CRP before the onset of the disease, that is, before the first mouse in the group developed CIA. Also, in addition to decreasing the incidence of CIA, CRP also delayed the progression of the disease by several days in both regimens.

### CRP does not reduce the severity of the disease

**Figure 4:**
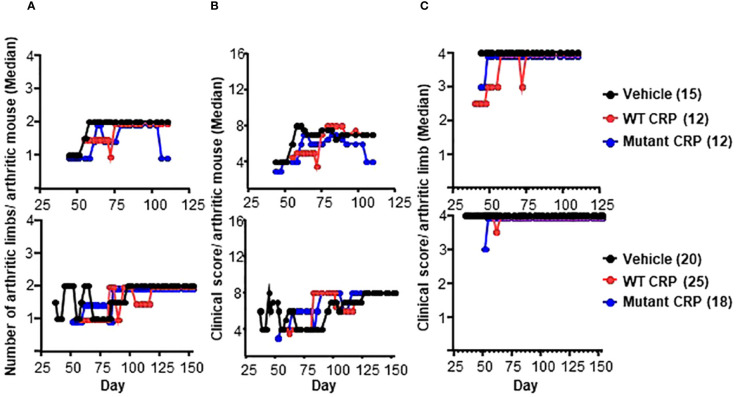
Severity of arthritis in mice from regimen 1 (top panels) and regimen 2 (bottom panels) experiments. **(A)** Median values for the number of arthritic limbs per arthritic mouse are shown. **(B)** Median values for the clinical score per arthritic mouse are shown. **(C)** Median values for the clinical score per arthritic limb are shown. The number of mice in each group is shown in the parentheses. To enhance the clarity of the graphs, in some panels, the numbers are not plotted as they were: For the vehicle groups, the numbers 1-4 are shown as 1-4. For WT CRP-treated groups, the numbers 1-4 are shown as 0.95, 1.95, 2.95 and 3.95. For mutant CRP-treated groups, the numbers 1-4 are shown as 0.9, 1.9, 2.9 and 3.9. There were no statistically significant differences between any two groups in all six panels (all *p* > 0.05).

The effects of CRP treatment on the severity of CIA were assessed by measuring the following three parameters: arthritic limbs/arthritic mouse, clinical score/arthritic mouse and clinical score/arthritic limb. The number of arthritic limbs/arthritic mouse ranged from 1 to 2 in all three groups of mice (**Figure 4A**). The clinical score/arthritic mouse ranged from 4 to 8 in all three groups of mice (**Figure 4B**). The clinical score/arthritic limb ranged from 2.5 to 4 in all three groups of mice (**Figure 4C**). There were no statistically significant differences in the severity of CIA between any two groups, for all of the three parameters, and for both regimens.

### CRP decreases the level of IL-17 and IL-6 but not TNF-α

**Figure 5:**
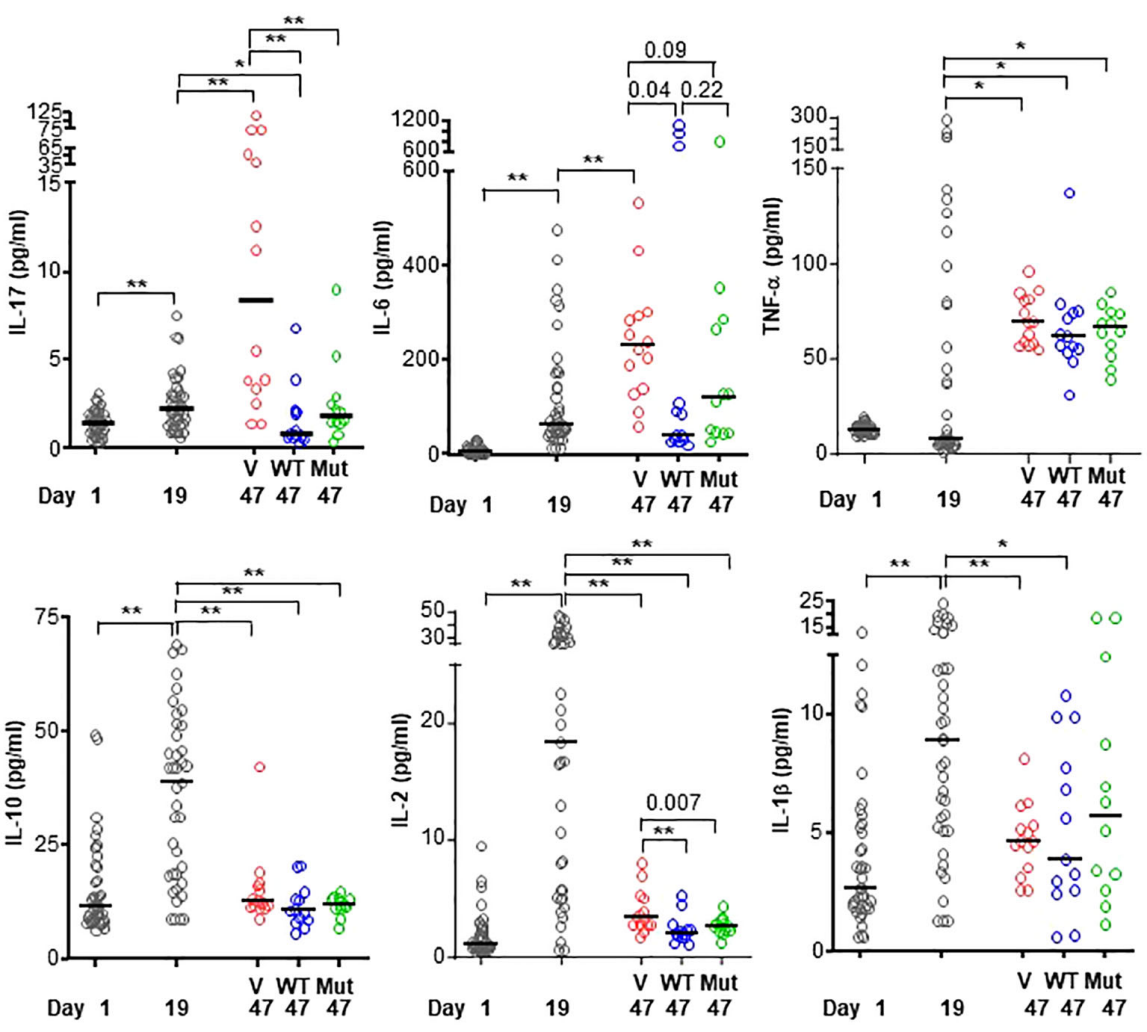
Levels of cytokines in the sera of mice from regimen 1 experiment. CIA was induced in 39 mice (black circles in all panels) on day 7. On day 26, mice were divided into three groups and treated with vehicle only (V; 15 mice; red circles in all panels), WT CRP (WT; 12 mice; blue circles in all panels) and mutant CRP (Mut; 12 mice; green circles in all panels). Sera were collected on day 1 (pre-immunization), day 19 (post-immunization and pre-CRP) and day 47 (post-CRP) for the measurement of cytokines. The median values of the concentrations of cytokines are shown. For clarity, *p* values are shown only for statistically significant differences (*p < 0.05, **p < 0.005); for some differences, the actual *p* values are shown.

Six of the cytokines produced by either phagocytic cells or T cells and which have been implicated in RA were measured (28, 44) in the sera of mice from regimen 1 experiment (**Figure 5**). The serum levels of all six cytokines increased in response to immunization and subsequent development of the disease, although the timing of appearance of each cytokine was different.

The profiles of IL-17 and IL-6 were similar. The serum levels of both cytokines increased until day 47, as shown in the vehicle group. However, in the CRP-treated groups, the levels of IL-17 and IL-6 did not increase. On day 47, levels of IL-17 and IL-6 were the same as on day 19, which was a day before the onset of the disease and before CRP adminstration began. There was no difference between the cytokine-reducing effects of WT and mutant CRP.

Like the serum profiles of IL-17 and IL-6, the level of TNF-α also increased until day 47, as shown in the vehicle group. However, unlike the effects of CRP on IL-17 and IL-6, CRP treatment did not prevent the increase in serum level of TNF-α. On day 47, the level of TNF-α was same as on day 19. There was no difference between the effects of WT and mutant CRP.

The profiles of IL-10, IL-2 and IL-1β were similar to each other but different from that of IL-17, IL-6 and TNF-α. The serum levels IL-10, IL-2 and IL-1β increased as early as day 19 and then returned to almost normal level by day 47, as shown in the vehicle group. CRP-treatment was not required to prevent the rise in levels of IL-10, IL-2 and IL-1β. Combined data on the effects of CRP on the serum levels of cytokines suggest that CRP reduces the levels of IL-17 and IL-6 but not of TNF-α, IL-10, IL-2 and IL-1β.

## Discussion

In this study, we investigated the interaction between CRP and agg-IgG and the effects of CRP on the development of CIA in mice. Our major findings were: 1. Immobilized IgG, whether it was mono-IgG or agg-IgG, expressed amyloid-like structures which could be detected by the antibodies to Aβ. 2. The Y40F/E42Q CRP mutant, which is in a non-native pentameric conformation, bound to immobilized IgG at physiological pH while WT CRP did so only at acidic pH. 3. CRP reduced the incidence of arthritis, that is, reduced the number of mice with developing arthritis. There was no difference in the protective capacities of WT and mutant CRP. 4. CRP did not affect the severity of disease in arthritic mice. 5. CRP decreased the serum level of IL-17, but not of TNF-α, in the CIA mouse model. There was no difference between WT and mutant CRP in preventing the rise in the serum level of IL-17.

Since ICs play a role in RA and in animal models of RA (45), interactions between CRP and ICs were evaluated, by employing agg-IgG. Agg-IgG is often used as a model of ICs (42). *In vitro* prepared ICs made up of any pair of antigen and antibody could not be used in the IgG-binding assays since WT CRP at acidic pH and mutant CRP at physiological pH were found to bind to both the antigen and the antibody when immobilized individually on microtiter plates (5, and data not shown).

Previous studies have also shown that WT CRP does not bind to either IgG or ICs, unless CRP was purchased commercially and used as it was (46, 47). However, CRP-IC complexes have been found *in vivo* (48, 49) and circulating ICs isolated from sera from patients with inflammatory diseases contained IC-complexed CRP (46, 47, 50–53). Also, similar to the binding of WT CRP to IgG at acidic pH and the binding of CRP mutant to IgG at physiological pH, mCRP has been shown to bind to IgG (20–22, 54) and binding of mCRP to IgG was increased at acidic pH (20–22). These earlier reports combined with our findings on CRP-IgG interactions suggest that the native pentameric conformation of CRP must be altered *in vivo* in order to bind to ICs. In the CIA murine model reported here, it was not clear whether CRP functioned in its native pentameric form or in its structurally altered pentameric form. The possibility can’t be ruled out that in the CIA mice WT CRP changed its structure *in vivo* after reaching the synovium and that’s why no difference was seen between WT and mutant CRP on their effects on the development of CIA.

The molecular mechanism of CRP-IgG interactions remains unclear. Previously, it has been shown that the binding of mCRP to IgG was differential and selective for different types of IgG (20, 22). It was hypothesized that the reason behind the differential binding of mCRP to various IgG was due to differential glycosylation of different IgG (46, 53). More recently, it was shown that the binding of WT CRP to immobilized IgG and various other protein ligands at acidic pH was also differential for different protein ligands (10). Similarly, the binding of CRP mutants to various immobilized protein ligands at physiological pH was found to be different for different protein ligands (5). Since immobilization of IgG and various other immobilized proteins to which CRP binds (5) results in the generation of amyloid-like structures, our data suggest that the binding of CRP to IgG was not due to the interaction between CRP and IgG *per se*, but due to the interaction between CRP and amyloid-like structures present on immobilized IgG. The extent of amyloid-like structures is different for various immobilized proteins (5). Accordingly, we hypothesize that the selective binding of CRP to various protein ligands including IgG is due to differential glycosylation and hence differential exposure of amyloid-like structures on various proteins.

The incidences of CIA in our *in vivo* experiments were 70% maximum in one experiment and 90% maximum in another regimen experiment. This rate of incidence is consistent with previously published reports where maximum incidences were 70%-80% (55–57). Since mutant CRP binds to IgG at physiological pH while WT CRP requires acidic pH to do so, we hypothesized that mutant CRP would be more protective against CIA compared to WT CRP. However, no differences were observed between WT and mutant CRP in reducing the incidence of arthritis. Also, the protective effects of CRP against arthritis were seen irrespective of whether there was only one injection or six injections of CRP before the onset of the disease, that is, before the first mouse in the group developed arthritis. Although CRP reduced the incidence drastically, the disease in the remaining arthritic mice was not less severe, indicating that CRP had effects on the onset of the disease but not on its subsequent clinical course.

By employing CRP-deficient mice and mice expressing human CRP transgene in CIA experiments in mice, it was concluded from the data that CRP exerts an early and beneficial effects on the development of arthritis (37, 38). However, the protection was due to the reduction in the severity of the disease. The reasons for the discrepancy in the results between these previously published studies (37, 38) and our study are not obvious. Not only in CIA, CRP has also been shown to alter immune responses in animal models of other autoimmune diseases including encephalomyelitis (58–63), antigen-induced arthritis (64) and nephritis (39, 65–67). In the case of encephalomyelitis, it has been shown that CRP was protective by suppressing both Th1 response directly and Th17 response indirectly (60). CRP has also been shown to have immunosuppressive functions in IC-induced immune cells (68).

IL-17 and TNF-α play crucial roles in the development of CIA (34, 57, 69). That serum levels of IL-17 and TNF-α increase in CIA mice has also been reported previously (57, 70–76). CRP did not significantly affect TNF-α levels in the CIA mice reported here, but *in vitro*, CRP has been found to inhibit the production of TNF-α in IC-induced monocytes (68). mCRP, however, increases the production of TNF from monocytes cultured *in vitro* (77). Our finding that CRP prevented rise in the serum level of IL-17 suggests that IL-17 is more critical than TNF-α for the initiation of the development of arthritis. This interpretation is supported by the findings that the deletion of the TNF-α gene does not confer complete protection from the occurrence of arthritis in CIA (78, 79) and that TNF-α and IL-17 act independently of each other under arthritic condition (80). In addition, when the effects of TNF-α inhibitors on IL-17 in patients with RA were evaluated, it was found that the beneficial effects of anti-TNF-α therapy might involve a decrease in IL-17 in responders (35).

IL-6 is also required for the development of CIA (55). Our finding that the serum level of IL-6 increases in the CIA model is consistent with previously published studies on IL-6 in CIA (55, 56, 70, 72–74, 76, 81, 82). We found that, like IL-17, CRP also reduced the serum level of IL-6. It has been proposed previously that IL-6 is more relevant than TNF-α in the development of CIA (79). Thus, both cytokines, IL-17 and IL-6, which are critical than TNF-α in arthritis, are inhibited by CRP. The profiles of IL-1β, IL-2 and IL-10 reported here are also consistent with the previously published data (27, 68, 70, 72, 73, 83) and CRP had no effect on these three cytokines.

Taken together, we conclude that CRP is an anti-arthritic molecule in this model system. This function of CRP involves the binding of CRP to ICs. IC-complexed CRP, through an as yet undefined mechanism, directly or indirectly, inhibits the production of IL-17 and eventually protects against the initiation of the development of arthritis. The finding that there was no difference between WT and mutant CRP in preventing the rise in the serum level of IL-17 suggests that CRP executes its functions in the synovium. Finally, the data suggest that IL-17, not TNF-α, is critical for the development of autoimmune inflammatory arthritis. It is also possible that CRP protects against autoimmunity in general, as has been hypothesized previously (84), by altering the levels of inflammatory cytokines *in vivo*.

## Data availability statement

The raw data supporting the conclusions of this article will be made available by the authors, without undue reservation.

## Ethics statement

Protocols approved by and conducted in accordance with the guidelines administered by the Institutional Animal Care and Use Committee of the Memphis VA Medical Center. The study was conducted in accordance with the local legislation and institutional requirements.

## Author contributions

SS: Data curation, Formal analysis, Investigation, Methodology, Writing – review & editing. AP: Data curation, Investigation, Writing – review & editing. DN: Data curation, Formal analysis, Investigation, Methodology, Writing – review & editing. UM: Data curation, Investigation, Writing – review & editing. AA: Data curation, Investigation, Writing – review & editing. DB: Data curation, Formal analysis, Investigation, Writing – review & editing. AA: Conceptualization, Formal analysis, Funding acquisition, Investigation, Supervision, Writing – original draft.
